# Direct Writing with Tilted-Front Femtosecond Pulses

**DOI:** 10.1038/s41598-017-13403-2

**Published:** 2017-10-10

**Authors:** Aabid Patel, Yuri Svirko, Charles Durfee, Peter G. Kazansky

**Affiliations:** 1 Optoelectronics Research Centre, University of Southampton, Southampton, SO17 1BJ United Kingdom; 2 Institute of Photonics, University of Eastern Finland, Joensuu, P.O. Box 111, FI-80101 Finland; 3 Department of Physics, Colorado School of Mines, Golden, CO 80401 USA

## Abstract

Shaping light fields in both space and time provides new degrees of freedom to manipulate light-matter interaction on the ultrafast timescale. Through this exploitation of the light field, a greater appreciation of spatio-temporal couplings in focusing has been gained, shedding light on previously unexplored parameters of the femtosecond light pulse, including pulse front tilt and wavefront rotation. Here, we directly investigate the effect of major spatio-temporal couplings on light-matter interaction and reveal unambiguously that in transparent media, pulse front tilt gives rise to the directional asymmetry of the ultrafast laser writing. We demonstrate that the laser pulse with a tilted intensity front deposits energy more efficiently when writing along the tilt than when writing against, producing either an isotropic damage-like or a birefringent nanograting structure. The directional asymmetry in the ultrafast laser writing is qualitatively described in terms of the interaction of a void trapped within the focal volume by the gradient force from the tilted intensity front and the thermocapillary force caused by the gradient of temperature. The observed instantaneous transition from the damage-like to nanograting modification after a finite writing length in a transparent dielectric is phenomenologically described in terms of the first-order phase transition.

## Introduction

For decades, spatio-temporal couplings in ultrafast optical systems have been relatively ignored and thought of as a hindrance since they typically arise from misaligned pulse compressors and dispersive optics^1–3^. However, recent developments in nonlinear microscopy and high harmonic generation have taken advantage of the interdependence of temporal and spatial coordinates of the beam^4^. A spatial separation of spectral components is conventionally used within pulse stretchers and compressors in chirped-pulse amplification, enabling high energies at short pulse durations to be reached without damaging the gain medium^5,6^. More recently, simultaneous spatial and temporal focusing (SSTF) has been utilized to suppress nonlinear effects outside of the Rayleigh zone by reducing the local bandwidth through the separation of the spatial components^4,7,8^. Bringing the separated components back together through focusing recovers the initial bandwidth and duration of the laser pulse, ensuring the maximum intensity at the focus. This eliminates self-focusing, improving the resolution in ultrafast laser material processing^9^ and nonlinear microscopy for imaging and surgical applications^10,11^.

A combination of spatial chirp (commonly characterized in terms of frequency gradient (*FRG*)) and angular dispersion (*AD*) can lead to a tilt of the pulse intensity front conventionally known as the pulse front tilt (*PFT*)^12,13^. The PFT has been found to being greatly beneficial for nonlinear frequency conversion with ultrafast lasers, particularly for travelling-wave pumping in X-ray lasers^14^, THz generation^15–17^ and achromatic phase matching^18,19^. Alongside the tilt of the pulse intensity front, there exists another manifestation of spatio-temporal couplings – a lighthouse-like rotation of the beam’s wavefront^20^. This coupling results in a change of the light propagation direction the pulse duration and is characterized in terms of the wavefront rotation (*WFR*)^21^. Recently the rotation of the beam’s wavefront has been utilized for angular separation of the successive attosecond pulses of high harmonics produced in laser plasma^22,23^. In ultrafast laser writing, the tilt of the pulse intensity front is thought to play a role in light-matter interaction with respect to asymmetries and polarization dependencies^24–29^. However, these past experiments were based on analysis of residual spatio-temporal couplings without any real characterization and/or control of the beam. With a greater appreciation of spatio-temporal couplings in focusing, it re-opens the discussion as to what spatio-temporal couplings play a role in ultrafast laser-material interactions.

In this paper, by comparing all major spatio-temporal couplings that reside in the pulse, we unambiguously show that in ultrafast laser writing, a finite PFT in the focus leads to an asymmetry with respect to the writing direction. We demonstrate that depending on the fluence and mutual orientation of the pulse intensity front and writing direction, the laser beam can produce either isotropic damage-like modification or birefringent nanograting structures in the focal area. We also observed an instantaneous transition from the isotropic damage-like modification to self-assembled nanogratings that can be explained in terms of the first-order phase transition. It should be noted the asymmetry with respect to the laser writing direction in transparent media discussed in this paper is different from the non-reciprocal laser writing^30^, which is writing in respect of reversing the light propagation direction in non-centrosymmetric crystals.

## Results

**Figure 1:**
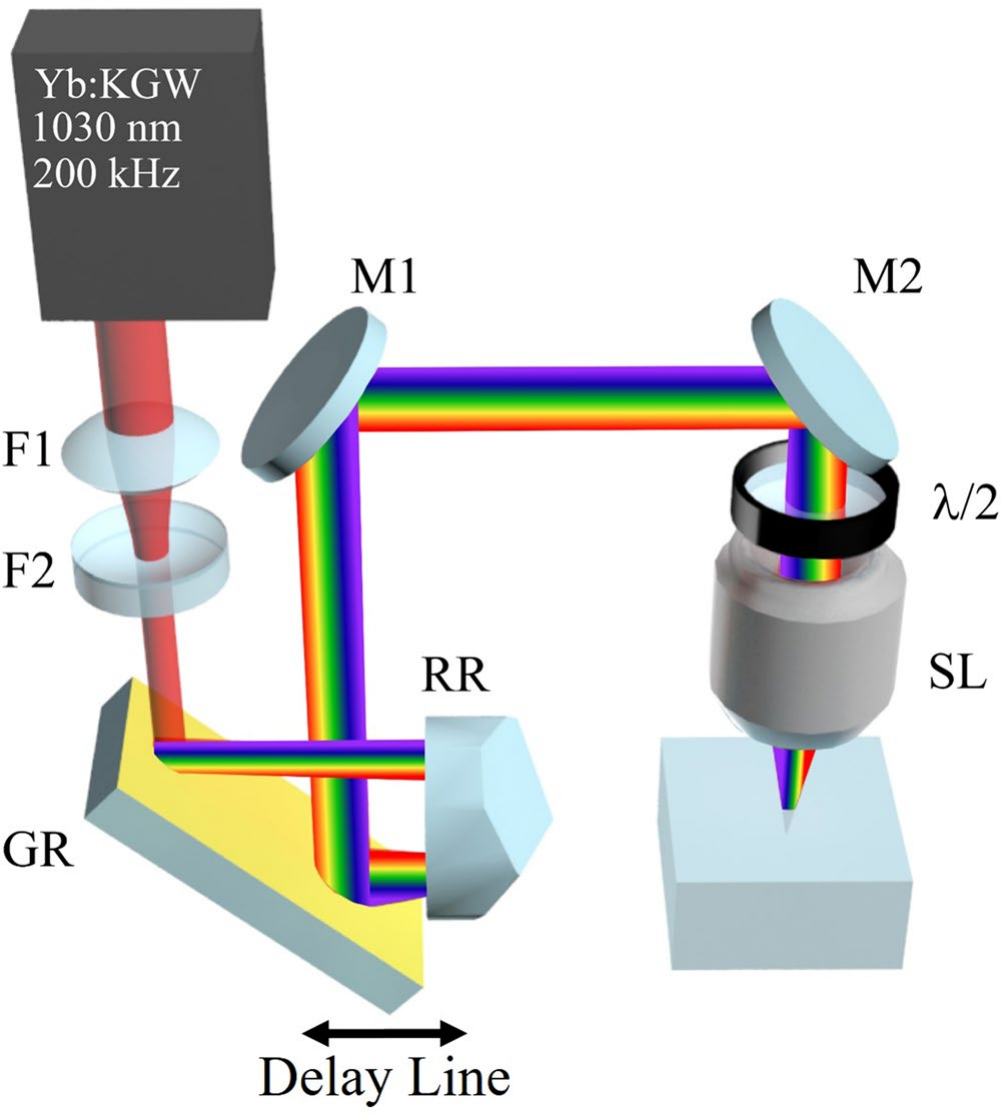
Sketch of the Spatio-Temporal Control set-up. The first grating compressor that controls temporal chirp is integrated with the Yb:KGW laser system. The second compressor enables control of spatial chirp by changing the distance between the grating (GR) and a retroreflector (RR). A Galilean telescope made of two lenses of focal distances F1 = 200 mm and F2 = −100 mm is placed before grating GR to ensure that the laser beam is not clipped by the focusing lens rear aperture. The use of the retroreflector ensures that there is no angular dispersion in the beam as it leaves the single-pass compressor (AD = 0). A wide-field SSTF focusing geometry ensures a circular beam spot near the focus, despite spatial chirp introduced by the second compressor. In the sketch of the setup, the M1 and M2 are dielectric mirrors, λ/2 is the half-waveplate and SL is the focusing lens.

Our experiments were carried out with an ytterbium doped potassium gadolinium tungstate (Yb:KGW) based mode-locked regenerative amplified femtosecond laser system PHAROS (Light Conversion Ltd.) emitting a train of femtosecond pulses at 1030 nm with 200 kHz repetition rate. Temporal chirp (*β*) and spatial chirp (FRG) were separately controlled with two grating compressors, allowing us to tailor the spatio-temporal properties of the beam (Fig. 1). The first compressor is incorporated into the laser cavity to control the temporal chirp by adjusting the group delay dispersion (*GDD*) with the use of a grating compressor in a double pass geometry. The second pulse compressor controlled spatial chirp, by varying the distance between a single grating and retro-reflector in a single-pass geometry. The second grating compressor was set to maintain a spatial chirp of FRG~1 nm/mm, resulting in a beam aspect ratio^31^ inside the sample of 1.52. The use of the retroreflector ensures that there is no angular dispersion in the beam as it leaves the compressor (*AD* = 0). Additionally, a Galilean telescope was placed before the second grating compressor to match the beam with the back aperture of the focusing lens. The beam coming from the laser was reduced by a factor of two to a 1/e^2^ diameter of ~4 mm.

The laser beam was focused with a 0.55 NA aspheric singlet lens 60 µm below the surface of a fused silica substrate, which was mounted onto a XYZ linear air-bearing translation stage (Aerotech Ltd.). It should be noted that with the beamlet diameter being ~4 mm, the focal spot size would be ~3 μm with a Rayleigh range of 9.9 μm inside the fused silica. This ensures that all material modification is inside the bulk of the fused silica rather than at the surface. The stage was computer controlled via SCA software (Altechna Ltd.) to translate the sample at uniform speeds. The polarization orientation was set to be either parallel or perpendicular to the spatial chirp with a λ/2 waveplate.

We studied the effect of the spatio-temporal couplings on the laser writing for pulses with PFT ~ ±0.6 fs/µm and PFT = 0, measured at the focusing optic. In order to achieve PFT of the same magnitude and different sign, the pulse is stretched to ~3 ps and then the sign of the temporal chirp was inverted (*β ~* ± 1.1 × 10^−6^ fs^−2^). To achieve zero PFT, the first (built-in) grating compressor was set to get *β* = 0, allowing the laser to produce a train of transform limited, spatially chirped 400 fs long pulses.

After the second compressor, the spatially chirped beam is then focused in a wide-field SSTF configuration, which yields a circularly symmetric focus despite ellipticity at the focusing lens caused by spatial chirp^32^. The pulse energies used in the laser writing experiments were 3.5 µJ (3.7 × 10^13^ W/cm^2^) and 2.5 µJ (2.7 × 10^14^ W/cm^2^) for the beam with PFT = ±0.6 fs/µm and PFT = 0, respectively.

### Sensitivity of Transition to Perturbations

**Figure 2:**
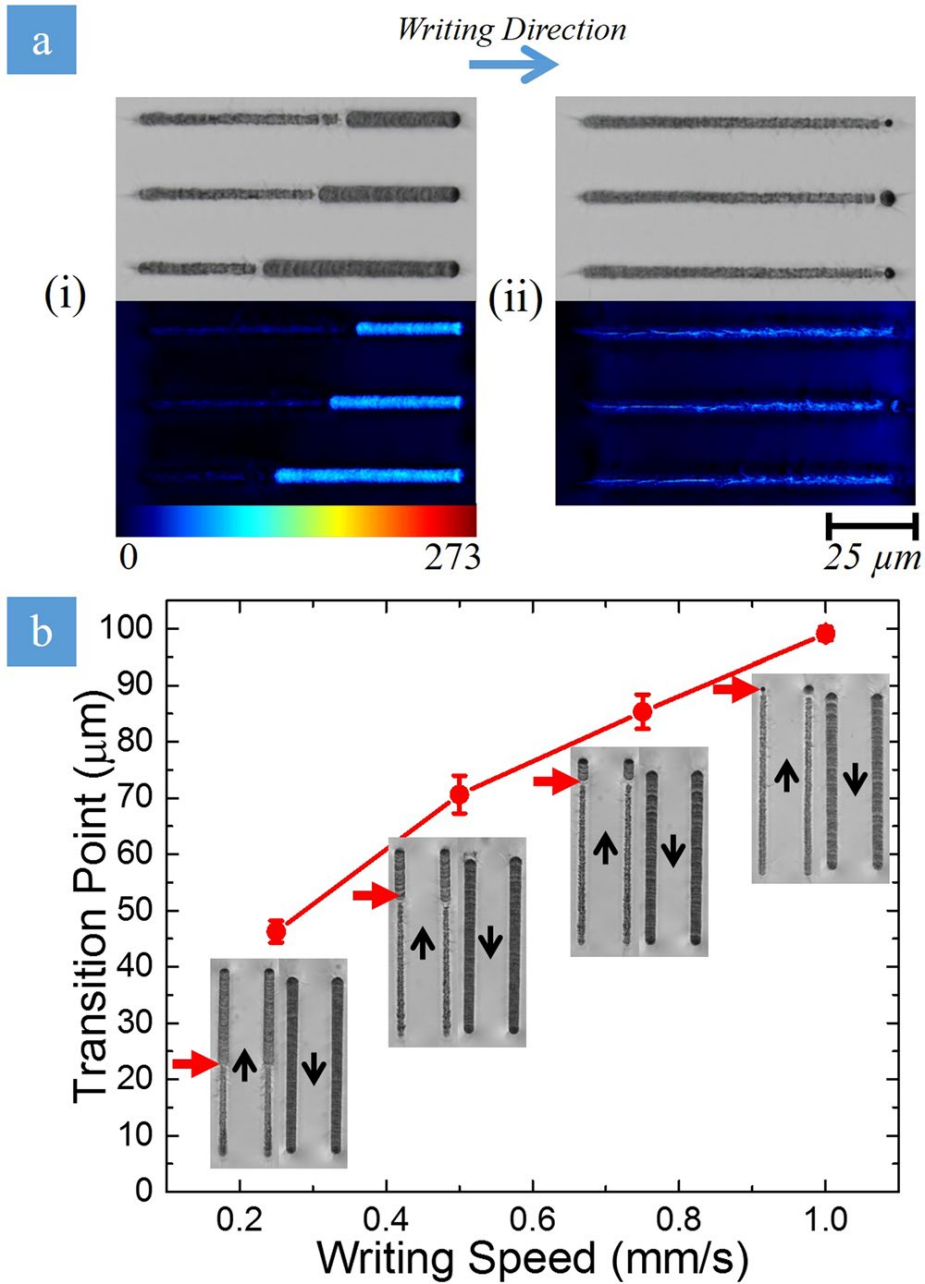
Transition from the damage-like state to nanograting modification. (**a**) Comparison of transition (i) with and (ii) without stage jitter (writing speed = 1 mm/s). The colour images are obtained with the quantitative birefringence measurement system (Cri Abrio, Olympus BX51), with the bright areas of the line representing the birefringent nanogratings (colour bar scale for retardance in nm). The regime transition occurs unpredictably in the vicinity of fluctuations during the writing process. When the fluctuations are removed, the transitions occur at the same point based upon writing speed and beam parameters. (**b**) The transition point dependence on writing speed. The transition between the damage-like modification and form birefringence (red arrows) occurs at a later distance and scales linearly with the writing speed. Nanograting formation is seen across the entire line when writing in the opposite direction (writing direction indicated by black arrows). Polarization is parallel to the writing direction in all cases.

A set of 100 µm long lines were written in the subsurface layer of the fused silica substrate by moving the sample at speeds from 0.3 mm/s to 1 mm/s at PFT = 0.6 fs/μm (Fig. 2). Our experiments revealed that the writing process is “asymmetric” in a sense that the silica glass was modified differently when we reversed the writing direction. Specifically in one writing direction, an isotropic damage-like modification with evidence of melting and crack formation abruptly changed to the formation of the birefringent nanograting structure after travelling a certain distance. However, no such change took place when the substrate was moving in the opposite direction. A drop in white-light emission is also observed at the point of transition during laser writing, indicating that the damage-like modification is associated with stronger white-light emission.

The transition between the two regimes (Fig. 2a(i)) is initiated by fluctuations during the laser writing process. In our experiments, these fluctuations arise in the jitter of the writing stages when insufficient time is given for the stage to stabilize after moving from one line to another. During laser writing, the laser turns on once the stages reaches the set writing speed. This entails particular acceleration and deceleration ramps, alongside the appropriate stops when changing from line to line. If the deceleration ramps and full stops are not set correctly, the stage does not have sufficient time to settle resulting in a stage jitter. As more lines are written, the jitter becomes more substantial resulting in the transition to occur earlier. Even with the appropriate acceleration ramps and stops, the transition can be initiated by vibrating the writing stage manually. It is worth noting that at the same writing speed, suppression of the fluctuations leads to the delay in the modification regime change (Fig. 2a(ii)).

After sufficient time to minimize the jitter is provided, the moment of the modification regime change is determined only by the beam parameters (laser fluence, intensity, etc.) and writing speed (Fig. 2b). The modification regime changes earlier at slower writing speeds when the beam moves in one direction (up arrow in Fig. 2b). On the contrary, for lines written by the beam moving in the opposite direction (down arrow in Fig. 2b), no transition is observed, i.e. nanograting forms from the start to the end of all the lines.

**Figure 3:**
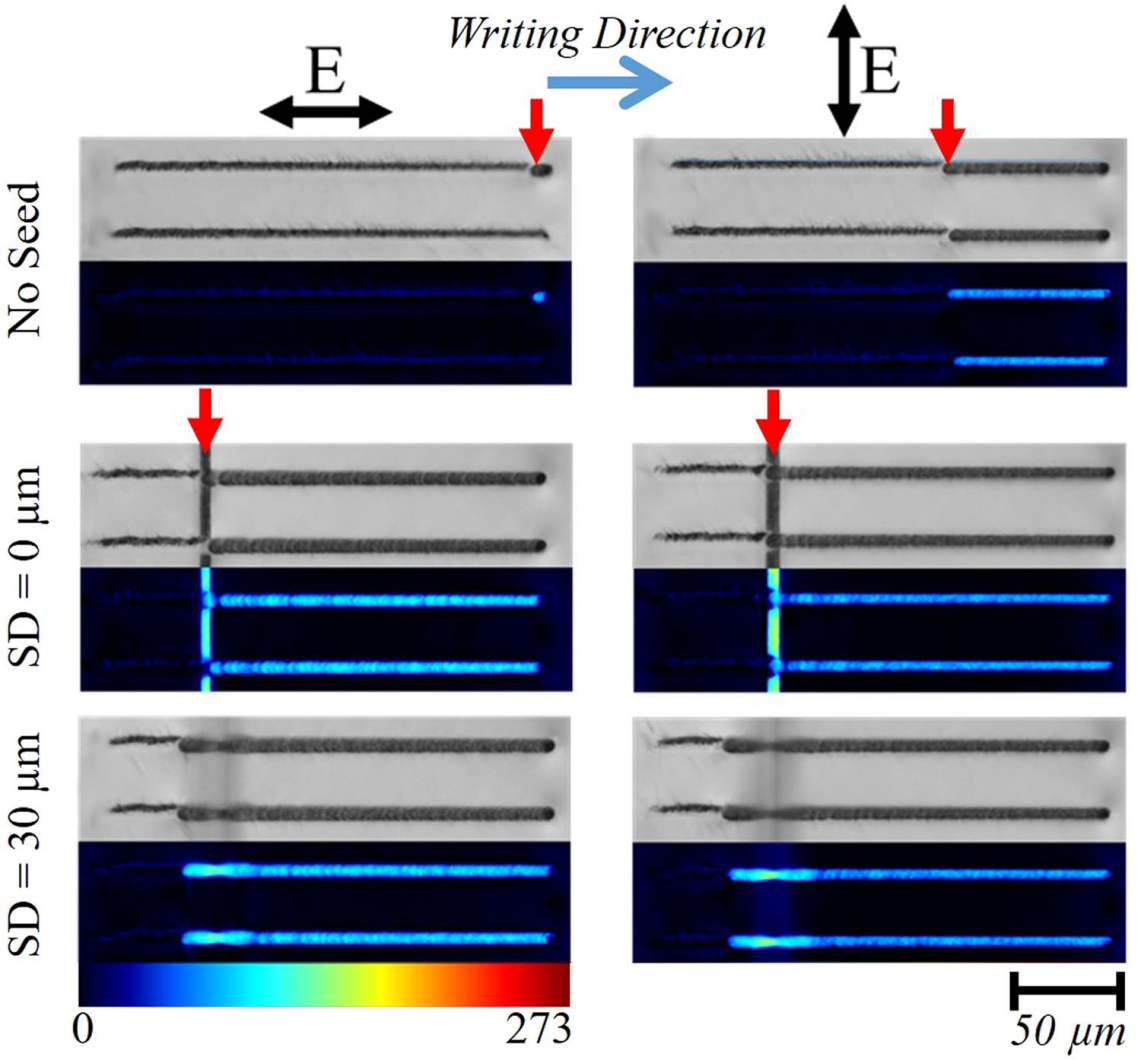
Instigating the damage-like modification to self-assembled nanograting transition with seed lines at different seed depths (SD). 200 µm length lines written at 1 mm/s with 3.5 µJ pulses. Regardless of the SD, the transition (red arrows) occurs when the laser line hits the pre-written seed, instigating the transition earlier than when there is no seed present. As the laser light interacts with the pre-written seed, the light is scattered effectively acting as a drop in pulse energy during laser writing. The drop in pulse energy experienced illustrates how minor fluctuations instigate the process.

In order to demonstrate that fluctuations instigate the change of the modification regime, “seed” tracks were written orthogonal to the writing direction. The scattering of the writing beam results in a sudden drop of fluence when the writing beam crosses the track (Fig. 3). When the seed track is not present, the modification regime transition does not occur or happens near the end of the line, depending on the beam polarization. When the seed is present, the transition occurs exactly where the beam crosses the track, regardless of the difference in depth between the seed line and written track or beam polarization. This indicates that a fluctuation in fluence caused by the scattering on the track is sufficient to instigate the change of the modification regime.

### Spatio-Temporal Couplings and the Directional Asymmetry of Writing

**Figure 4:**
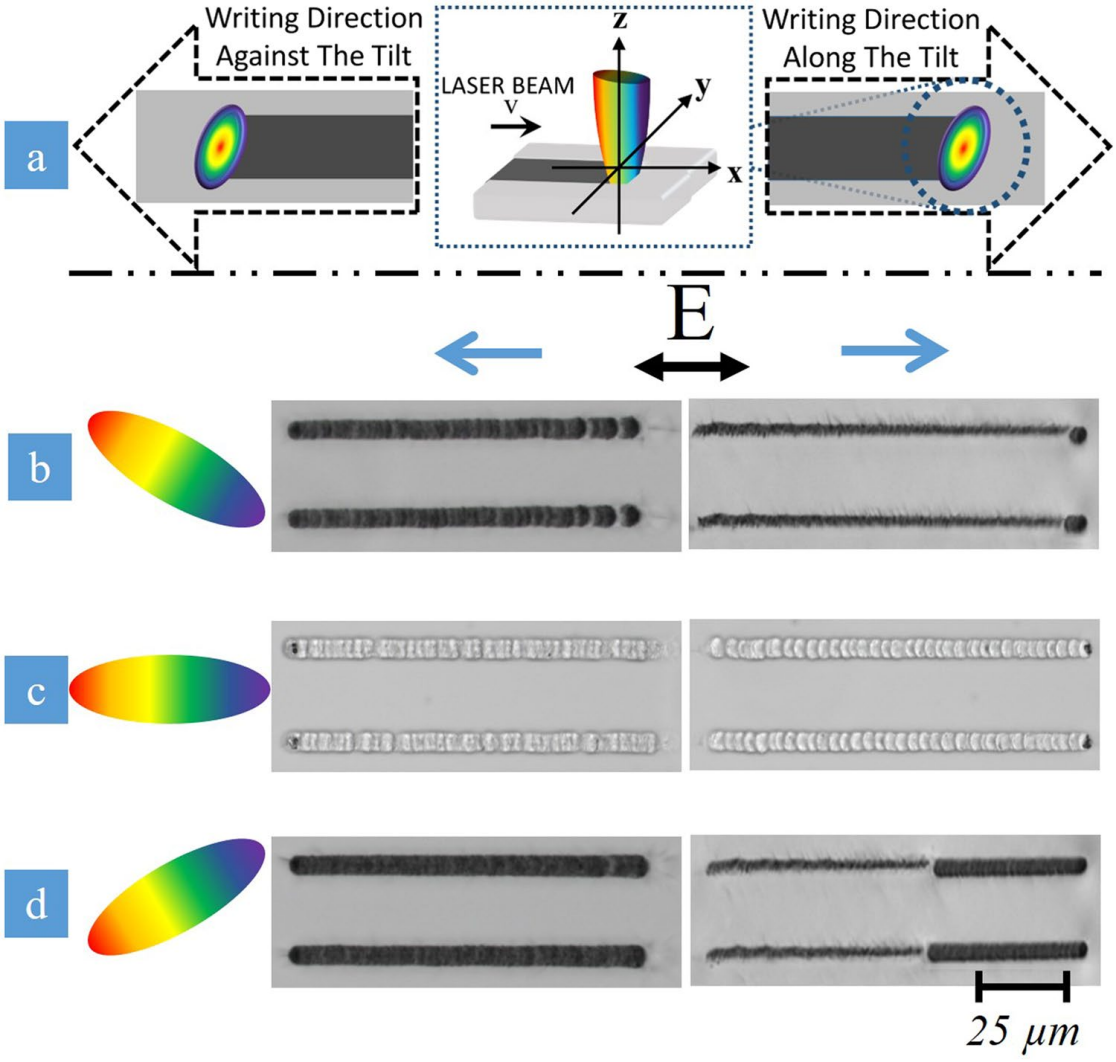
Directional dependence on writing for 3 different spatio-temporal coupling cases (Writing Speed = 1 mm/s). (**a**) Schematic diagram indicating orientation of PFT with respect to writing direction (black dashed arrows), depicting how lines are inscribed in the direction along and against the tilt (i.e. parallel (+x) and anti-parallel). (**b**) Negative *β* (PFT = 0.6 fs/μm, 3 ps, pulse energy is 3.5 µJ). Directional dependence is observed with nanograting formation when moving along the tilt and damage-like modification transitioning to the nanograting state in the opposite direction. (**c**) *β* = 0 (PFT = 0, 400 fs, 2.5 µJ). No directional dependence is evident with non-uniform nanograting modification in both directions. (**d**) Positive *β* (PFT = −0.6 fs/μm, 3 ps, 3.5 µJ). The directional dependence of writing does not reverse with the change in PFT orientation, even though the quill-effect disappears when PFT = 0. All PFT cases for (**b**–**d**) are schematically shown for the PFT conditions before the lens.

The dependence of the writing asymmetry on the spatio-temporal coupling was studied by comparing optical microscope images of the lines inscribed in the bulk of the fused silica at positive, negative and zero temporal chirp, by writing along and against the tilt direction (Fig. 4a). The temporal chirp was varied using the first (internal) grating compressor while keeping the second grating compressor untouched to ensure the same FRG. Directional dependence is clearly observed when temporal chirp is negative (i.e. PFT > 0 before the lens). Nanograting formation is observed when writing was along the tilt direction while the damage-like modification is observed when the writing is against the tilt direction (Fig. 4b). At zero temporal chirp (i.e. PFT = 0 before the lens), no discernible directional dependence is evident, confirming previous studies^29^ (Fig. 4c). When temporal chirp is positive and PFT < 0, the directional dependence re-emerges but does not flip with the change in sign of PFT as anticipated^24,25^ (Fig. 4d). Specifically, isotropic damage-like modification occurs when writing along the tilt direction and nanograting formation is seen when writing against the tilt direction. The directional writing asymmetry is originally thought to flip with the change in sign of PFT before the lens^24,25^, which contradicts the observations made.

It is worth noting that both femto- and picosecond pulses induce comparable birefringence. However, the modified area created at longer pulse durations is observably darker when viewed under the optical microscope. Specifically, transmission measurements using a VIS microspectrophotometer (CRAIC) revealed 90–95% and 28–39% transmission yielded from femto- and picosecond pulses, respectively. Such a remarkable difference originates from the scattering and the development of stress regions surrounding the lines incurred with picosecond pulses^33^ and is important to take into account for the fabrication of optical devices, such as radial waveplates, when minimum loss upon transmission is required^34^.

## Discussion

The observed directional dependence of writing not flipping with the tilt (Fig. 4d) contradicts previous results^25,29^, thus re-opening the discussion on the origin of this effect. In previous studies reporting on this phenomenon, the sign of the PFT was changed either by holograms^29^ or by adding an additional mirror to the beam path^25^. However, both of these methods either changed the orientation of the spatial chirp along with the angular dispersion or simply flipped the entire beam with all associated spatio-temporal couplings. Thus, it is not surprising that in both cases, the directional dependence of writing was observed to flip with PFT. In contrary, we employ two independent compressors to control the magnitude and direction of pulse front tilt, without affecting the spatial chirp before the lens. This approach allows us to visualize the role of the different spatio-temporal couplings (e.g. wavefront rotation, the tilt of the intensity front, etc.) in femtosecond laser writing and reveal the origin of the observed directional asymmetry.

In order to describe spatio-temporal couplings in the Gaussian laser pulse propagating along the z-axis, it is convenient to present the electric field in the following form^20^:

1 $$E(x,t)\propto \exp \{{\tilde{Q}}_{xx}{x}^{2}+2{\tilde{Q}}_{xt}-{\tilde{Q}}_{tt}{t}^{2}\}$$

where the y-dependence of the electric field is suppressed. The complex parameters ${\tilde{Q}}_{xx}=-1/{a}^{2}-ik/2R$ and $\,{\tilde{Q}}_{tt}=1/{\tau }^{2}-i\beta$, where *a* is the beam spot size, *k* is the wavevector, *R* is the wavefront curvature, *τ* is the pulse duration, and *β* is the temporal chirp, describe the spatial and temporal characteristics of the beam, respectively. GDD is also determined by ${\tilde{Q}}_{tt}$, i. e.

2 $$GDD=\frac{1}{2}\,{\rm{Im}}\,[\frac{1}{{\tilde{Q}}_{tt}}]=\frac{\beta {\tau }^{4}}{2({\beta }^{2}{\tau }^{2}+1)}$$

One can observe from Eq. (2) that while GDD and *β* are representative of the same quantity for Gaussian pulses, conversion from one to the other requires the pulse length.

The spatio-temporal couplings are represented by the complex parameter ${\tilde{Q}}_{xt}$ in Eq. (1). The experimental observables can then be presented as the following^35^:

3 $$AD={\rm{Re}}\,[\frac{{\tilde{Q}}_{xt}}{{\tilde{Q}}_{tt}}]$$

4 $$FRG=\frac{2\,{\rm{Im}}[{\tilde{Q}}_{xt}\cdot {\tilde{Q}}_{tt}^{\ast }]}{{\rm{Re}}[{\tilde{Q}}_{tt}]}$$

5 $$PFT=\frac{{\rm{Re}}[{\tilde{Q}}_{xt}]}{{\rm{Re}}[{\tilde{Q}}_{tt}]}$$

6 $$WFR={\rm{Im}}[{\tilde{Q}}_{xt}]$$

It is worth noting that in the Gaussian pulse with spatial and temporal chirp, the experimentally observable parameters are dependent on one another. In particular, by combining Eqs (2–6), the following relations can be found:

7 $$PFT=(FRG\times GDD)+AD$$

8 $$WFR=\frac{1}{2}FRG-\beta \times PFT$$

Thus, in order to control the propagation direction of the wavefront (i.e. WFR) and the intensity front (i.e. PFT) in the beam, it is necessary to suppress angular dispersion in the beam. At *AD* = *0*, Eqs (7) and (8) becomes:

9 $$PFT=\frac{FRG\times \beta {\tau }^{4}}{2(1+{\beta }^{2}{\tau }^{4})}$$

10 $$WFR=\frac{FRG}{2(1+{\beta }^{2}{\tau }^{4})}$$

Therefore, the direction of the WFR follows the FRG, while the sign of PFT depends on both FRG and *β*.

One can observe from Eqs (9 and 10) that the PFT and WFR do not change sign under reversal of the writing direction because the spatial chirp (i.e. the value of FRG) remains unchanged^20^. However, the magnitude of WFR decreases with PFT. When *β* = 0, *WFR* = *FRG/2* is at a maximum, but directional dependence of writing is not observed (Fig. 4c). In contrast, the directional dependence is observed when the WFR before the lens is slower (Fig. 4b,d). Specifically, when *β ~* 1.1 × 10^−6^ fs^−2^, FRG = 1 nm/mm and PFT = 0.6 fs/µm, the WFR over the beamspot is approximately 0.05 mrad/ps (7.85 × 10^7^ revolutions per second). When *β* = 0, the WFR is approximately 0.3 mrad/ps (4.7 × 10^8^ revolutions per second). The physical rotation of the wavefront during the pulse is 0.09 mrad and 0.165 mrad at *β ~* 1.1 × 10^−6^ fs^−2^ and *β* = 0, respectively. These values are a few orders lower than the WFR being utilized in attosecond generation and streaking^22,23,36^, by exploiting the lighthouse-based movement in plasma. It should be noted that in contrast to our experimental set-up (Fig. 1), in previous studies WFR was induced due to angular dispersion of a large bandwidth source before the lens, resulting in the direction and large magnitude of WFR to be only dependent on angular dispersion^23^.

**Figure 5:**
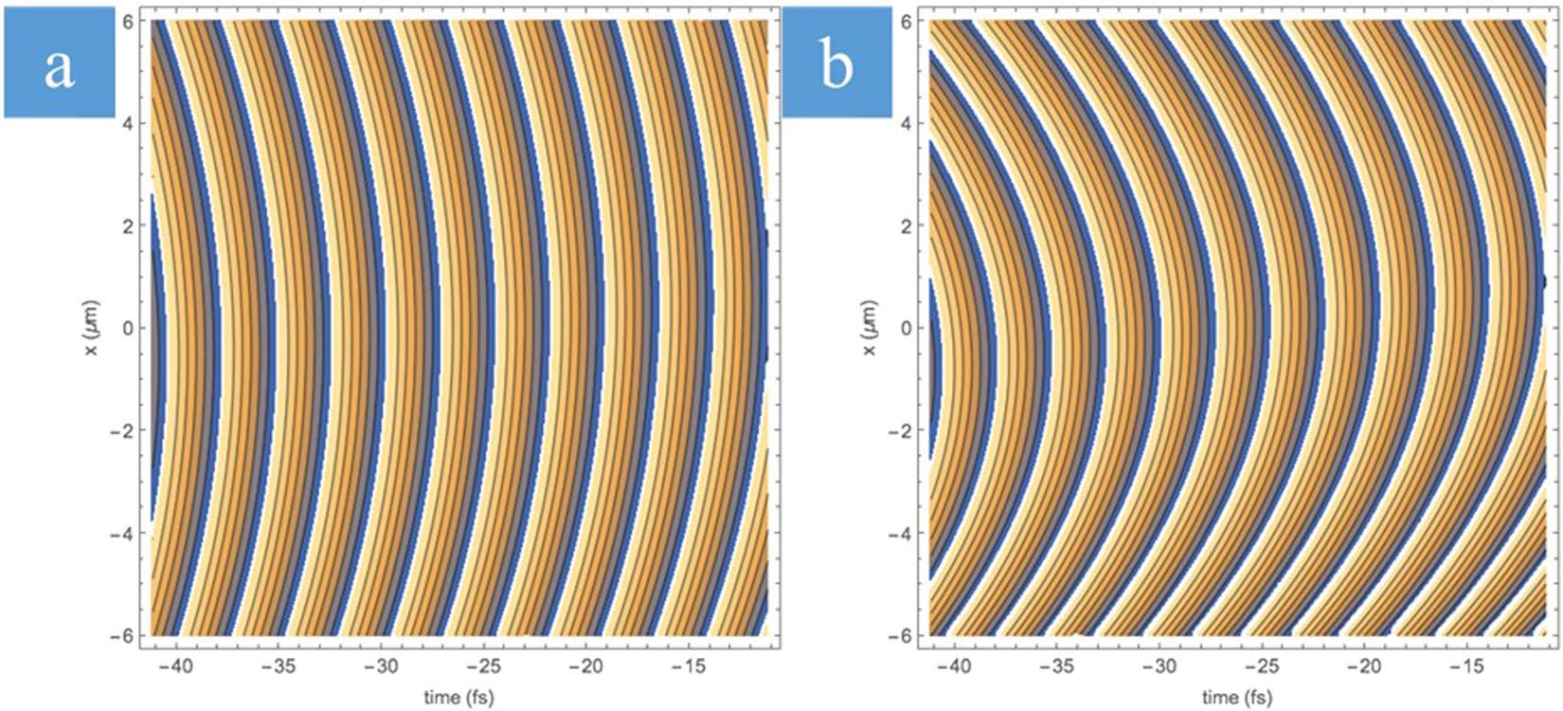
Contour plots of wavefronts (Im[log[E(x, t)]) at axial position z = z_R_/2 (z_R_ is the Rayleigh range). (**a**) GDD = 700 fs^2^. Each frequency component arrives at a different time, so this curvature matches a Gaussian CW beam description. (**b**) GDD = 0. Frequency components are superimposed and the angular variation of the beam with spatial chirp enhances the wavefront curvature. Pulse duration is set to 15 fs for the sake of illustrating the wavefronts.

It is worth noting that WFR would still be dominated by FRG before the depth of focus making the direction of rotation unchanging in the focused beam. In order to illustrate this, simulations were performed for a 15 fs pulse (we considered a short pulse duration to enhance the effect) (Fig. 5). When plotting the contours of the phase along focusing, it can be seen that for large values of *β* (GDD = 700 fs^2^), the arrival of the spectral components is sheared out in time similar to what would be observed for CW Gaussian beams (Fig. 5a). When *β* = 0 (GDD = 0), the curvature of the wavefronts is enhanced from the angular variation of the different spectral components (Fig. 5b). In the context of the experimental conditions, the enhancement would be much smaller at 400 fs and even smaller at 3 ps, effectively being sheared out in time. This allows us to conclude that in our experimental conditions, rotation of the wavefront during the pulse unlikely affects the inscription process in the bulk silica glass. Therefore, WFR cannot be the origin of the writing asymmetry effect making PFT the remaining spatio-temporal coupling, which could cause the directionally asymmetric writing.

**Figure 6:**
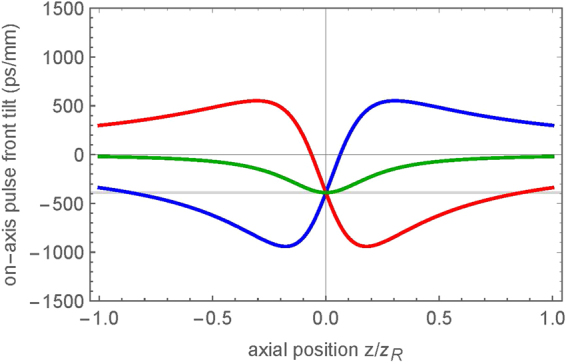
Evolution of PFT along focusing for the three different PFT cases. In the case of positive PFT before the lens (red line), the PFT maximizes as the beamspot shrinks making FRG maximum before the focal plane of the lens. At the focal plane, the spectral components overlap making FRG = 0. Angular dispersion dependent PFT dominates at the focus (PFT ≈−200 fs/μm). In the case of zero GDD (and thus zero PFT before the lens, green line), the PFT remains small along the optical axis except near the focal plane where the PFT depends only on the AD. For the negative PFT before the lens case (blue line), the evolution is similar to the positive case but flipped along the focal plane.

It is important to understand that PFT is affected by focusing^32,37,38^. Recently it has been shown that regardless of the state of a spatially chirped beam before the lens, the intensity front will always be tilted at the focus^31,39^ and is dependent on the spatial chirp at the lens entrance and the focal beam waist^31^. Using the formalism developed in ref.^25^, we performed the analysis of the evolution of PFT along the propagation direction in and out of the focal plane, as a function of GDD (Fig. 6). One can observe from Fig. 6 that for when PFT > 0 before the lens (red line, GDD ~ 4.5 × 10^5^ fs^2^), PFT increases as a function of the axial position along focusing. To understand the non-monotonic dependence of the PFT on the axial position, one can recall that since AD = 0 before the lens, *PFT* = *FRG* × *GDD* (Eq. (7)), i.e. tilt of the intensity front is due to the combination of spatial and temporal chirp. Focusing results in a finite AD and the spectral components begin to overlap as the beam spot shrinks. This increases the FRG and hence both terms in Eq. (7) contribute to the PFT, which grows rapidly. However, once the beam reaches the Rayleigh range of the focusing optic (depth of focus), the beamspot no longer shrinks as the spatial components continue to overlap. At the focus, FRG = 0 and the second term in Eq. (7) dominates resulting in *PFT* ≈ *AD* ≈ −200 fs/μm. For the PFT < 0 case (blue line, GDD ~ −4.5 × 10^5^ fs^2^), the evolution of PFT is flipped at the focal plane. When PFT is zero before the lens (green line, GDD = 0), it remains zero out of the Rayleigh range but at the focus the PFT due to angular dispersion becomes evident, maximizing in magnitude at the focus to the same value (*PFT* ≈ −200 fs/μm) as seen in the cases with non-zero GDD.

**Figure 7:**
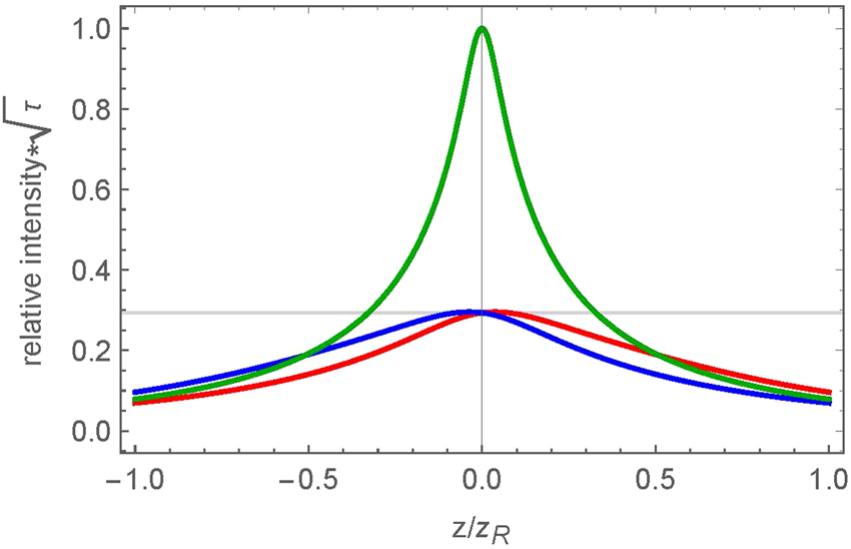
Relative intensity threshold evolution during focusing for the three PFT cases. The zero PFT case (green line) is larger by a factor of 3 than the non-zero PFT cases (PFT > 0 – red line, PFT < 0 – blue line). The grey line indicates where the non-zero PFT cases reach their max intensity and threshold for modification. The zero PFT case reaches that same threshold at z_R_ in front of the focus where PFT is minimal. To reach the same threshold at the focus, the pulse energy for the zero PFT case must be decreased by a factor of three. The location of maximum intensity for the non-zero PFT cases are slightly shifted from the focus, depending on the sign of the temporal chirp.

Therefore in our experimental conditions, both the zero and non-zero PFT before the lens cases result in the same PFT in the focal area. The question arises why the asymmetry in laser writing *was observed* at non-zero PFT (Fig. 4b,d) and *was not observed* at zero PFT (Fig. 4c), alongside why the directional dependence *does not flip* when PFT changes sign. To explain this, we need to take into account that in our experimental conditions, at the laser fluence in the focal plane of about ~10^2^ J/cm^2^, the maximum focal intensities are ~10^2^ TW/cm^2^ and ~10^3^ TW/cm^2^ at non-zero and zero PFT, respectively. This drastic difference in intensity indicates that modification of the silica at zero and non-zero PFT takes place at *different* distances from the focal plane. Comparing the relative intensities for the different PFT cases as a function of the square root of the pulse duration (Fig. 7), the PFT = 0 case (Fig. 7, green line) reaches the same intensity as the non-zero PFT cases (Fig. 7, red and blue lines) before the focal zone (~z_R_). Thus, the beam with zero PFT before the lens enables ionization and material deformations at z_R_ where the PFT during focusing remains close to zero (Fig. 5, green line). In this situation, the intensity front is perpendicular to the beam axis and no directional asymmetry in the laser writing may emerge when *β* = 0.

When PFT before the lens is non-zero, the modification would take plane near the focal plane (i.e. the picosecond pulses deposit energy closer to the focus^33^.) One can observe from Fig. 6 that PFT in the focal plane is independent on the sign of *β*, being entirely due to the angular dispersion from the lens. Therefore, the inscription of the lines written within the bulk of the fused silica takes place at the same PFT ≈ −200 fs/μm (Fig. 5). This explains why the asymmetric writing behavior does not flip when the sign of PFT before the lens changes.

It is worth noting that in order to observe directional dependence at *β* = 0, the intensity at the focal plane needs to be similar to that seen for the non-zero PFT cases (i.e. pulse energy decreased by a factor of 3). However at such low intensities, nanogratings may form provided the writing speeds are low but the damage-like modification would not occur, making it difficult to observe the asymmetrical writing behavior.

It should be noted that propagation non-linearities are not implemented within the modelling of the PFT evolution in focusing. Based on the threshold for non-linear propagation through calculation^33^, all experiments are conducted in an environment where focusing is dominated by the lens (i.e. there is no self-focusing). While there is no evidence of any self-focusing or other propagation non-linearities, there may potentially be some slight non-linearities in the focus due to the large intensities reached. Past studies have discussed how spatio-temporal couplings change in focusing due to sample dispersion and other phase abberations^32,40^. However, these changes are only apparent at relatively short pulse durations (i.e. τ ~ 100 fs). In our experimental conditions, these slight changes would not drastically alter the spatio-temporal properties of the pulse from what is seen in our analysis (Figs 6 and 7).

**Figure 8:**
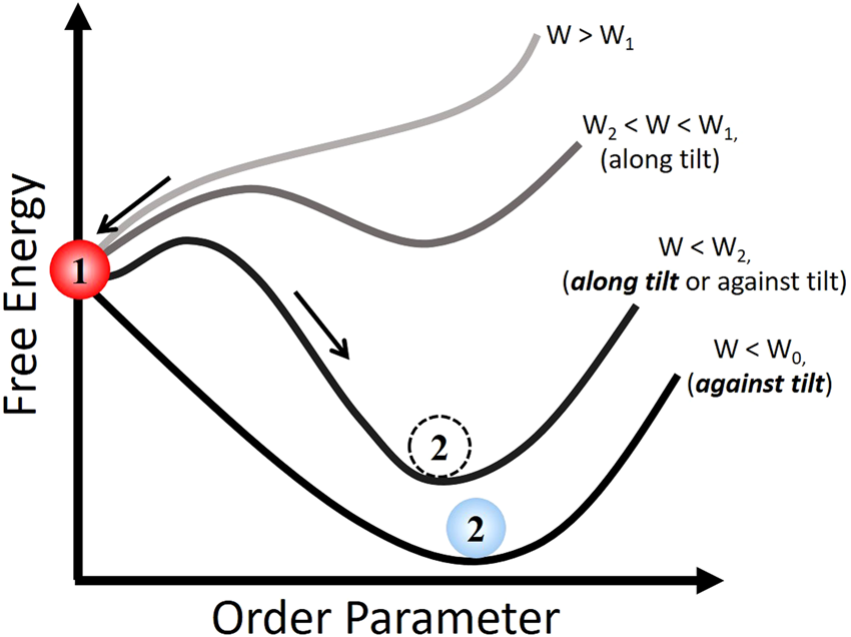
First-order phase transition interpretation for the switching of the modification regime from an isotropic damage-like state (1) to the nanograting state (2). When the absorbed energy, *W*, exceeds the damage-like state threshold, *W*_1_, the damage-like state corresponds to the absolute minimum of the free energy (i.e. the writing produces the damage-like modification of the substrate). When *W*_*2*_ < *W* < *W*_*1*_, the damage-like state at η = 0 is energetically favorable. However, there is a local minimum of the free energy at η = η_1_, which is associated with the metastable nanograting state. When the absorbed energy decreases further *W* < *W*_2_, the absolute minimum of the free energy corresponds to the grating state. In such a case, the damage-like state becomes metastable. That is a fluctuation in the writing process can add an activation energy necessary to switch from the isotropic damage-like to the birefringent nanograting state. At *W* < *W*_0_, the free energy has only a minimum, which corresponds to the formation of the nanograting.

The observed switching of the modification regime from the formation of the isotropic damage to the formation of the nanograting when writing and tilt direction coincide (Fig. 4b,d) is a very interesting scientific finding. It can be thought of in terms of a first-order phase transition^41^ (Fig. 8) where latent heat is needed to initiate the transfer from one material phase to another. Using this analogy, the switching of the modification regimes can be seen as a phase transition in the modified region between an isotropic damage-like state (Fig. 8, (1)) and a nanograting state (Fig. 8, (2)). Following the Landau theory, we can introduce the free energy of the modified region as the following:

11 $$F={a}_{1}{\eta }^{2}+{a}_{2}{\eta }^{3}+{a}_{3}{\eta }^{4}$$

Here *η* > *0* is the order parameter, which describes the form-birefringence in the modified region and *a*_*1*_, *a*_*2*_ and *a*_3_ depend on the energy, writing speed and other beam parameters. In the experiment, it is natural to assume that the absorbed energy, *W*, of the laser pulse determines the state of the modification. In the framework of the Landau theory, we can assume that *a*_*1*_ = *α*(*W* − *W*_*0*_), where *α* > 0 is constant and *W*_*0*_ determines the absorbed energy that corresponds to the stable state with *η* > *0* (i.e. form birefringence in the modified region). The theory of the first-order phase transition states that at high intensities (*W >> W*_*0*_), *η* = 0 is the only solution that minimizes the free energy^41^ (Eq. (11)). Moreover, when *a*_*2*_ > *0*, there are no other positive *η* to satisfy the equation.

12 $$\frac{\partial F}{\partial \eta }=2{a}_{1}\eta +3{a}_{2}{\eta }^{2}+4{a}_{3}{\eta }^{3}=0$$

when *a*_2_ < *0*, another solution of Eq. (12) that corresponds to the minimum of the free energy is permitted:

13 $${\eta }_{1}=\frac{1}{8{a}_{3}}(3|{a}_{2}|+\sqrt{9{{a}_{2}}^{2}-32{a}_{3}\alpha (W-{W}_{0}))}$$

This solution exists at *W* < *W*_1_ where:

14 $${W}_{1}={W}_{0}+\frac{9{{a}_{2}}^{2}}{32{a}_{3}\alpha }$$

However, this solution, which represents the damage-like state, is unstable until the free energy is positive (i.e. the free energy exceeds the free energy of the damage-like state, *F*(*η*_1_) > *F*(*η*_0_) = 0). When the absorbed energy decreases down to:

15 $${W}_{2}={W}_{0}+\frac{{{a}_{2}}^{2}}{4{a}_{3}\alpha }$$

the free energy of the damage state eventually becomes equal to that of the nanograting state. In other words, the first order transition between the damage and birefringent state occurs. As intensity decreases further, the nanograting state becomes metastable and finally, at *W* = *W*_*0*_, there is no local minimum of the free energy at η = 0 (damage-like state is forbidden).

In our phenomological phase transition model of the ultrashort pulse modification, the absorbed energy is equivalent to temperature in the Landau phase transition theory. For writing along the tilt, when the absorbed energy is higher than writing against the tilt, we observe the metastable damage-like state converted into the nanograting state when an activation energy is supplied to the system (i.e. via fluctuation in the incident fluence). Remarkably, the transition takes place without a change in the absorbed energy. This is similar to the freezing of supercooled water that is not accompanied with a change in temperature. By further exploring this analogy with respect to the damage-like to nanograting switching and the first order phase transition, there should be an analog of latent heat, which is released into the system similar to the water freezing process. This “latent heat” in the femtosecond laser writing process may be associated with the energy consumed in the formation of nanovoids in the damage-like state or released as potential energy of the electrons clustering in the formation of nanogratings. The latter is supported if one assumes that white light accompanying the modification originates from the thermal emission of electrons or Bremsstrahlung emission. Correspondingly, the drop in white-light emission observed in the nanograting regime may indicate that the kinetic energy of electrons is converted into potential energy associated with the charge separation (clustering) of the plasma producing the nanograting.

It is worth noting that the observed switching from the damage-like to nanograting modification is different from the transition and self-healing process of nanogratings that is dependent on the randomness of crack initiation in glass^42^. This transition can revert back from one state to another simply by continuing the writing process, while the transition observed in our experiment is irreversible.

**Figure 9:**
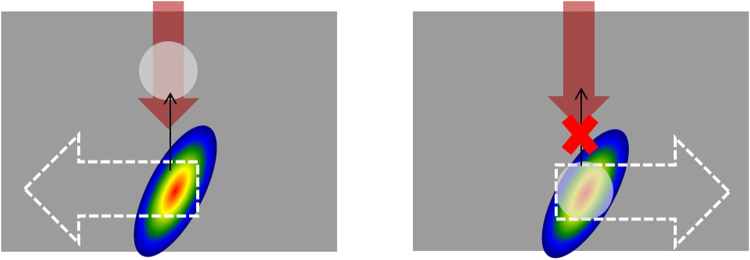
Schematic diagram of potential void-trapping mechanism for the directional asymmetric writing effect. During the writing process, voids are formed initially and are subsequently trapped by a combination of forces. If the beam translation is against the tilt (left), thermocapillary forces trap the void in the horizontal direction. However, the void could move towards the head of the structure limiting the energy deposition due to scattering and promoting nanograting growth. If the beam translation is along the tilt (right), the void is again trapped by thermocapillary forces but is also forced to stay near the focus by the gradient of the tilted intensity front. This allows the following pulses to interact with pristine material, allowing for stronger energy absorption. As writing continues, the instantaneous transition from the damage-like state to the nanograting state occurs after a noted incubation length. This transition could be the result of the void growing to a critical size or a generated charge from dragging a trapped void where it would scatter the incoming laser light after the noted incubation length, triggering the transition.

The directional asymmetry in laser writing was originally interpreted based on an anisotropic trapping of electron plasma due to a ponderomotive force^24,25^, when the pulse front is tilted in the writing direction. The issue with this explanation is the need of large intensities at the focal plane to observe an effective “snow-plow” displacement of electrons^43–45^, which are thought to be difficult to reach in the material due to a laser induced plasma clamping effect^46^. However, it has been determined that in tight focusing regimes (NA > 0.4), voids appear within the first few pulses^47–49^. The voids eventually transition into self-assembled nanogratings after further irradiation^47^. Evidence in previous publications suggest that voids could be potentially created within the first few pulses and then trapped or constantly collapsing and reforming during the writing process, resulting in a void at the end of an inscribed line^25,50–52^ (Fig. 9). The formed void could interact with the intensity front differently depending on the orientation of the tilt. We suggest that two different forces govern the behavior of this void during laser writing. The first one is the gradient force in the tilted front that could push the void out of the region of high intensity. The second is a thermocapillary force, which pushes the void into the region of high temperature and intensity (Marangoni effect)^53^. When the laser writing is in the direction against the tilt (Fig. 9 left), the thermocapillary force traps the void in the horizontal direction, confining it in the high temperature region. As writing continues, the void could move towards the head of the structure, increasing the scattering and promoting nanograting growth. When the laser writing is in the direction along the tilt (Fig. 9 right), the void is again formed near the focus of the beam and is trapped in the horizontal direction by the thermocapillary force. As the writing continues, the gradient force from the tilted intensity front keeps the void from moving vertically (i.e. gradient force > thermocapillary force), forcing it to stay near the focus. This allows the following pulses to remain unperturbed, interacting with pristine material to ensure efficient energy deposition. Writing in this direction produces the damage-like modification, which results in a metastable, “supercooled” state of modification. As the writing length increases, an instantaneous transition from the damage-like to nanograting modification takes place. This indicates that the seed necessary for nanograting growth could be produced after a certain incubation length, as seen by the dependence of the transition point as a function of the writing speed (Fig. 2). In other words, writing along the tilt results in a continuous change of an unknown (or “dark”) parameter, which is not revealed in the appearance of modification. We speculate that this “dark” parameter could be the result of the void increasing in size or a generated charge as the laser writing continues. The void could continue to grow as more pulses are deposited into the bulk while the void is trapped within the focal region. The void would reach a critical size where it could either overcome the forces trapping it and allowing the void to move towards the head of the structure or the void could explode/collapse. With respect to the charge scenario, the charge could be produced by diffusion of electrons from the region of high intensity in the focus of the beam^54,55^ or dragging of the weakly trapped void similar to an electrostatic charge generated by flowing liquids^56^. The charge would accumulate to a critical value as writing length increases until it reaches the governed incubation length when it promotes the void and nanograting formation. These scenarios allow the critically sized void or generated charge to interfere with the laser irradiation where it would incur scattering and instigate the transition to nanograting formation. However, the noted incubation length associated with the transition and what causes and governs it is still up for debate.

Therefore, the conditions for strong energy absorption could be achieved by controlling the PFT of ultrashort laser pulses and writing in the direction along the tilt. Previous laser writing experiments have stated that typically 40–60% of the laser energy is consumed from absorption, reflection and scattering depending on the experimental conditons^33,49^. We speculate based on the thresholds for damage-like modification, writing along the tilt direction provides a 5–15% increase in energy absorption as opposed to writing against the tilt direction, which can be proven useful in the manufacturing of optical elements using ultrafast laser writing. However, it depends on the application whether it is advantageous to laser write with a tilted intensity front. If there were a need for uniform modification of the same strength/state regardless of direction, then it would be advantageous to laser write in a regime where there is zero PFT. One way to ensure this is by laser writing within the bulk where it is expected that PFT is zero by choosing the appropriate intensities, similar to the zero PFT case in experiment. Another way is to remove all spatio-temporal couplings of the beam before the focusing optic. While conceptually straightforward, in practice it is difficult to work in a regime of zero PFT. Any laser that has a built-in chirped pulse amplification design can produce residual spatio-temporal couplings due to slight misalignments^27^, which can lead to strong asymmetries in writing. In order to choose the appropriate laser intensity where PFT is zero, a thorough understanding of the spatio-temporal properties is necessary. Since the identity of these spatio-temporal couplings can be quite complicated (i.e. spatial chirp, angular dispersion, higher-order dispersion, etc.), it is difficult to characterize effectively. To correct for them, complicated diffractive element-based designs of SLM methods are necessary. Alongside that, the concept of SSTF in laser writing is to produce stronger modification closer to the focus, which would result in PFT at the focus^27,57^. Therefore, it might be advantageous to use PFT as an extra degree of freedom in direct laser writing. By understanding the mutual orientation of the PFT with respect to the writing direction, one can utilize the PFT inherent in their bare beam to create stronger modification by writing in the appropriate direction.

## Conclusion

Here we demonstrated unambiguously that the tilted pulse front is the origin of the asymmetry of the direct ultrafast laser writing with respect to the reversal of the writing direction. We have confirmed that the switching between modification regimes can be manipulated via the control of spatio-temporal couplings of the laser beam and that the directionally asymmetric laser writing follows the direction of the tilt of the intensity front in the area where modification occurs. Our analysis shows that the switching from damage-like modification to self-assembled nanogratings can be qualitatively described in terms of the first-order phase transition. The noted incubation length observed for the transition to take place could be explained in terms of voids trapped within the focus of the laser beam and affected by a combination of the gradient of the tilted intensity front and thermocapillary forces. However, the exact mechanism of this phenomenon is still an open question. Knowing SSTF is pivotal in creating highly localized modification, the same associated spatio-temporal couplings will lead to the manifestation of the asymmetric orientational writing effect, which is vital to control and enhance ultrafast laser material processing.

## Methods

Our experiments were carried out with an ytterbium doped potassium-gadolinium tungstate (Yb:KGW) based mode-locked regenerative amplified femtosecond laser system PHAROS (Light Conversion Ltd.) emitting a train of femtosecond pulses at 1030 nm with 200 kHz repetition rate. Temporal chirp and spatial chirp were separately controlled with two grating compressors, allowing us to tailor the spatio-temporal properties of the beam (Fig. 1). The first compressor is incorporated into the laser cavity to control the temporal chirp by adjusting the group delay dispersion (*GDD*). The second pulse compressor controlled spatial chirp, by varying the distance between a single grating and retro-reflector. The second grating compressor was set to maintain a spatial chirp of ~1 nm/mm, resulting in a beam aspect ratio^31^ of ~1.9 before focusing. Owing to refraction entering the sample, the effective beam aspect ratio is reduced inside to 1.52. The amount of residual spatial chirp introduced by the first compressor was negligible, being roughly ten times smaller. Angular dispersion is ensured to be zero in the beam along the entire propagation length before the lens. Additionally, a Galilean telescope was placed before the second grating compressor to match the beam with the back aperture of the focusing lens. The beam coming from the laser was reduced by a factor of two to a 1/e^2^ diameter of ~4 mm.

The laser beam was focused with a 0.55 NA aspheric singlet lens 60 µm below the surface of a fused silica substrate, which was mounted onto a XYZ linear air-bearing translation stage (Aerotech Ltd.). It should be noted that with the beamlet diameter being ~4 mm, the focal spot size would be ~3 μm with a Rayleigh range of 9.9 μm inside the fused silica. This ensures that all material modification is inside the bulk of the fused silica rather than at the surface. The stage was computer controlled via SCA software (Altecj12hna Ltd.) to translate the sample at uniform speeds. The polarization orientation was set to be either parallel or perpendicular to spatial chirp with a λ/2 waveplate.

In order to achieve PFT of the same magnitude and different sign, the pulse is stretched to ~3 ps yielding PFT at the focusing optics with a magnitude of ~±0.6 fs/µm by switching the sign of *β* (*β ~* ±1.1 × 10^−6^ fs^−2^). To achieve zero PFT, the first (built-in) grating compressor was set to get transform limited, spatially chirped ~400 fs long pulses (*β* = 0). PFT was estimated using Eq. (7) and experimentally measured using the FRG, in terms of frequency (dω/dx), and the GDD to attain units of fs/μm.

The spatial chirp was characterized by measuring the FRG with an optical fiber coupled spectrometer. The temporal chirp was monitored with an APE pulseCheck multi-shot autocorrelator. Measured values were confirmed by the Kostenbauder matrix formalism^58^.

The spatially chirped beam is focused, resulting in SSTF. While the spatial component separation is the key aspect towards SSTF, the imaging geometry used upon focusing is also crucial to define. There are two forms of SSTF: wide-field scanning and line-scanning. Both of these forms are based upon how the beam diameter falls on the focusing optic. In the wide-field scanning, the beam shape is elliptical and spatially chirped along the elliptical axis. This would mean that in one direction, the beam is a thin line where the different spectral components are spread out, and in the other direction, the beam is effectively monochromatic. It should be noted that in this geometry, the effective NA of the lens would be reduced since each monochromatic beam will not focus tightly. This results in a spherically symmetric beamspot at the focus as all the spectral components recombine. In line-scanning SSTF, the beam is spatially chirped in one dimension but has a circular beam spot at the lens. This results in each monochromatic portion of the beam to focus tightly, yielding an elliptical beamspot at the focus. In this study, the beam is focused in a wide-field SSTF configuration, which yields a circularly symmetric focus despite ellipticity at the focusing lens caused by spatial chirp^32^. The pulse energies used in experiment were 3.5 µJ (3.7 × 10^13^ W/cm^2^) and 2.5 µJ (2.7 × 10^14^ W/cm^2^) for the beam with PFT = ±0.6 fs/µm and PFT = 0, respectively.

**Figure 10:**
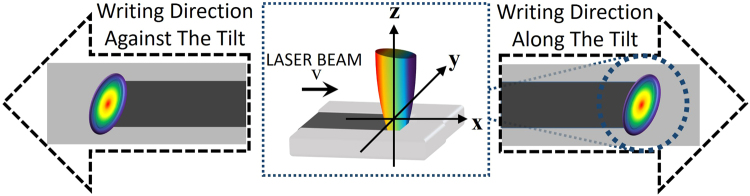
Schematic diagram indicating orientation of PFT with respect to writing direction (black dashed arrows). Lines are inscribed in the direction along and against the tilt (i.e. parallel (+x) and anti-parallel (−x)). Polarization was set to be either parallel to the writing direction (x) or orthogonal to the writing direction (y). The propagation direction, z, is normal to the surface of the material.

Sets of 100 µm long lines were written in the subsurface layer of the fused silica substrate by moving the sample at speeds from 0.3 mm/s to 1 mm/s for the different PFT cases studied (Fig. 10). In each case, the sample was translated either along, *x*, or against, *−x*, the tilt of the pulse intensity front, while setting the laser polarization to be parallel, *x*, or orthogonal, *y*, to the writing direction. The laser propagation direction, *z*, is normal to the sample’s surface.

### Data accessibility

All data in this paper was deposited into the University of Southampton Library Repository (doi:10.5258/SOTON/D0259).
